# Serum Amyloid A as a Marker of Persistent Inflammation and an Indicator of Cardiovascular and Renal Involvement in Patients with Rheumatoid Arthritis

**DOI:** 10.1155/2014/793628

**Published:** 2014-11-27

**Authors:** Bożena Targońska-Stępniak, Maria Majdan

**Affiliations:** Department of Rheumatology and Connective Tissue Diseases, Medical University of Lublin, ul. Jaczewskiego 8, 20-950 Lublin, Poland

## Abstract

*Objectives*. Rheumatoid arthritis (RA) is a systemic, inflammatory disease. Serum amyloid A (SAA) is an acute-phase protein, involved in pathogenesis of atherosclerosis. The aim of the study was to assess serum concentration of SAA in RA patients, with reference to other inflammatory parameters and markers of extra-articular involvement. *Methods*. The study population consisted of 140 RA patients, low/moderate disease activity (L/MDA) in 98 (70%) patients and high disease activity (HDA) in 42 (30%). Comprehensive clinical and laboratory assessment was performed with evaluation of electrocardiogram and carotid intima-media thickness. *Results*. The mean SAA concentration [327.0 (263.4) mg/L] was increased highly above the normal value, even in patients with L/MDA. Simultaneously, SAA was significantly higher in patients with HDA versus L/MDA. The mean SAA concentration was significantly higher in patients treated with glucocorticoids, was inversely associated with QTc duration, and was markedly higher in patients with atherosclerotic plaques, emphasizing increased CV risk. SAA was significantly higher in patients with increased cystatin-C level. *Conclusions*. In RA patients, high serum SAA concentration was strongly associated with activity of the disease and risk of CV and renal involvement. Recurrent assessment of SAA may facilitate searching patients with persistent inflammation and risk of extra-articular complications.

## 1. Introduction

Rheumatoid arthritis (RA) is a progressive, autoimmune disease, characterized by chronic inflammation of synovial membrane and consistently degradation of articular cartilage and subchondral bone.

Serum amyloid A (SAA) is a highly conserved acute-phase protein, released in response to inflammation or infection. Production of acute-phase SAA (A-SAA) is stimulated by proinflammatory cytokines, such as interleukin-6 (IL-6), Il-1, tumor necrosis factor (TNF), interferon-*γ*, and transforming growth factor-*β* (TGF-*β*). The concentration of A-SAA increases dramatically during acute inflammation and injury, reaching within 5-6 hours levels that are 1000-fold greater than normal [1–3]. As with other acute-phase reactants, the liver is the primary source of circulating A-SAA. However, extrahepatic production of SAA by several tissues and cell types has been described in patients with chronic diseases, for example, Alzheimer's disease, cancer, diabetes, obesity, insulin resistance, metabolic syndrome, and atherosclerosis [4–6]. SAA is accepted as an adipokine which directly mediates obesity-related inflammation [4]. It has been even suggested that SAA may play an important, pathogenic role in the proinflammatory cascade in the course of RA [6].

SAA is not only an acute-phase protein but also a kind of apolipoprotein, relevant in cholesterol metabolism. Normally, SAA circulates in low levels bound to high-density lipoprotein (HDL), but during inflammation SAA can contribute up to 80% of HDL apolipoprotein (apo) composition, exceeding apo-A1 in quantity and impairing the protective function of HDL. SAA may also increase the oxidation of low-density lipoprotein (LDL) and thus may be associated with cardiovascular (CV) disease (CVD) and atherogenesis [5, 7].

SAA is also the precursor of amyloid A protein, a fibrillar, insoluble product deposited in major organs, which carries a risk of organ failure and premature death in the course of secondary amyloidosis. AA amyloidosis is a late complication of RA, connected with active, long-standing, and disabling disease. The prevalence of AA amyloidosis in RA reported in different studies is highly variable because the complication frequently remains undetected due to lack of clinical manifestations in many patients [8]. Persistently high concentration of SAA is a prerequisite for AA amyloidogenesis [1, 3]. High levels of SAA correlate with the progression of amyloidosis and low levels are associated with its regression [2, 9]. It was reported that, in AA amyloidosis patients, serum SAA concentration was the most important predictive parameter of the risk of death, associated with renal function [8].

The aim of the study was to assess the concentration of SAA in RA patients with different disease activity, with reference to other inflammatory, metabolic parameters, as well as CV and renal disease risk factors.

## 2. Materials and Methods

### 2.1. Study Population

The study group consisted of 140 RA patients admitted consecutively and treated in the Department of Rheumatology and Connective Tissue Diseases, Medical University of Lublin. The patients were qualified for the treatment in order to make a decision whether to maintain or modify the ongoing therapy, depending on an assessment of the disease activity. All patients fulfilled the American College of Rheumatology criteria for the classification of RA [10]. Informed consent was obtained from the patients according to the Declaration of Helsinki. The Ethical Committee of the Medical University of Lublin approved the design of the study. Demographic and clinical information was obtained through structured interview, review of medical records, self-report questionnaires, physical examination, laboratory tests, and high-resolution B-mode ultrasonography.

### 2.2. RA-Related Data Collection

Disease activity was measured using the Disease Activity Score (DAS) based on evaluation of 28 joints (DAS28), calculated with the number of tender joint counts (TJC) and swollen joint counts (SJC), erythrocyte sedimentation rate (ESR) value, and patient's global assessment (PGA) of disease activity in visual analogue scale (VAS) [11]. Ability to perform daily activities was measured using the Modified Health Assessment Questionnaire (M-HAQ) with the range 0–3 (a score 0 representing no impairment of function) [12]. Erosive form of RA was diagnosed in those patients, who presented erosions on joint surfaces of bones in radiograms of hands and/or feet, according to Steinbrocker et al.'s criteria [13]. Extra-articular symptoms were noted, which occurred during the whole course of the disease.

### 2.3. Laboratory Tests

Blood was collected after an overnight fasting to determine the complete blood cell count, erythrocyte sedimentation rate (ESR), serum concentration of CRP, creatinine (Cr), total protein, albumin, fibrinogen, total cholesterol (TC), HDL-cholesterol [HDL-C], LDL-cholesterol [LDL-C], and triglycerides (TG) at the University Hospital central laboratory. Serum level of CRP was measured by immunoturbidimetric assay, with the upper limit of the normal range at 5 mg/L. Concentrations of TC, HDL-C, and TG were measured using the standard enzymatic technique (BIOMAXIMA); LDL-C was calculated according to the Friedewald formula. Modification of diet in renal diseases (MDRD) was calculated for every patient to estimate the glomerular filtration rate (GFR) using serum Cr concentration and demographic factors [14].

Blood samples were also stored at −80°C for further assessment of cystatin-C (Cys-C) and SAA. Cys-C is used as an endogenous marker for GFR, more precise than creatinine [14]. Serum Cys-C was measured using quantitative enhanced immunonephelometry method (with commercially available assay developed by DADE Behring). The producer recommended the normal range of Cys-C between 0.53 and 0.95 mg/L independently of sex, age, and body mass. Serum concentration of SAA was determined by a commercial enzyme-linked immunosorbent assay (ELISA), with detection limit 0.005 mg/L (Human SAA; BioSource Europe S.A., Belgium). According to literature, SAA normal reference range is under 10 mg/L [15].

Assessment of RA serological markers was performed. IgM-rheumatoid factor (RF-IgM) was determined using the antirheumatoid factor ELISA (IgM) test (EUROIMMUN) with the recommended upper limit of the normal range 20 units (RU)/mL. Anticitrullinated protein antibodies (ACPA) were determined using the QUANTA Lite CCP3.1 IgG/IgA ELISA assay (INOVA Diagnostics) with negative results <20 U/mL.

### 2.4. Assessment of CV Parameters and Risk Factors

Height and weight were measured barefoot wearing light clothes. Body mass index (BMI) was calculated as the ratio of weight and squared height. During physical examination blood pressure (BP) was assessed in a sitting position. The standard 12-lead transthoracic electrocardiogram (ECG) was performed in every patient, with assessment of corrected QT interval (QTc). Measurement of QTc was performed automatically.

The 10-year risk of fatal CVD using the Systemic Coronary Risk Evaluation (SCORE) model according to the EULAR recommendations was estimated in every patient [16].

Carotid intima-media thickness (cIMT) was measured using high-resolution B-mode ultrasound (Logiq 7 GE). In every subject IMT was assessed bilaterally in the three regions: common carotid artery (CCA), carotid bulb (BULB), and internal carotid artery (ICA). The average of the maximal IMT from all 6 carotid segments (defined as mean cIMT) was used in the analyses. It has been reported that cIMT ≥ 0.6 mm is a marker of subclinical atherosclerosis [17]. The presence of carotid plaques is a marker of advanced atherosclerosis. Plaques were defined as a distinct protrusion, greater than 1.5 mm into the vessel lumen [18].

### 2.5. Statistical Analysis

Results were expressed as mean (standard deviation, SD) or number (%). Variables were tested for normality by Kolmogorov-Smirnov's test. Group differences were tested using Student's *t*-test and Mann-Whitney *U* test for normally and nonnormally distributed parameters, respectively. Spearman's or Pearson's correlation test was used to determine the association between SAA and clinical and laboratory variables. Multivariable analysis (multiple linear regression) was performed according to a forward selection procedure, introducing those variables that showed a statistically significant association with SAA. For all tests, *P* values < 0.05 were considered significant.

## 3. Results

### 3.1. Demographic and Disease-Related Variables in RA Patients

Characteristics of patients with RA are presented in Table 1; clinical and laboratory variables are presented in Table 2. Most patients were positive for RF-IgM and ACPA and had erosive form of RA, of low or moderate activity (L/MDA) (DAS28 ≤ 5.1) at the time of assessment. Remission of RA according to DAS28 (<2.6) was observed in 8 patients (7 women and 1 men). Extra-articular symptoms during the course of RA were noted in 58 patients (41.4%) and included rheumatoid nodules (40 patients), sicca syndrome (16 patients), interstitial lung disease (7 patients), amyloidosis (1 patient), and vasculitis (1 patient). At the time of examination disease modifying antirheumatic drugs (DMARDs) were not used in 5 patients (3.6%). In the remaining 135 patients, treatment with at least 1 synthetic DMARD was administered: methotrexate (MTX) (58.6% of all patients), leflunomide, sulfasalazine, chloroquine, and cyclosporine. Biological DMARDs were used in 36 patients (25.7%) (adalimumab in 3, etanercept in 15, infliximab in 15, and rituximab in 3 cases). Simultaneously, low-dose prednisone (≤10 mg/day) was used in 108 patients (77.1%).

### 3.2. Assessment of SAA Concentration in Patients with RA

The mean SAA concentration in the group of 140 patients was 327.0 (263.4) mg/L and was associated with parameters of disease activity and inflammation. Positive, significant (*P* < 0.05) correlations were found between SAA and DAS28, TJC, SJC, PGA of the disease activity, morning stiffness, M-HAQ and ESR value, concentration of CRP, fibrinogen, and Cys-C, as well as with white blood cell count (WBC), platelet count (PLT), and SCORE value. Negative, significant (*P* < 0.05) correlations were found between SAA and concentration of albumin, hemoglobin, and QTc value. All the above-mentioned variables were included in the multiple linear regression analysis, which confirmed significant associations for CRP, WBC, and QTc (Table 3).

### 3.3. Evaluation of SAA Concentration in Distinct Groups of RA Patients

The mean SAA concentration was significantly higher in men than in women (*P* = 0.01) (Table 4). The group of men, when compared with women, was characterized by higher inflammatory parameters [CRP 27.4 (23.3) versus 17.4 (21.9) mg/L, *P* = 0.005; fibrinogen 5.4 (1.2) versus 4.6 (1.3) g/L, *P* = 0.006], unfavorable lipid parameters [HDL 50.7 (11.6) versus 62.3 (15.0) mg/dL, *P* = 0.0001; TC/HDL index 3.8 (1.0) versus 3.4 (0.8), *P* = 0.01], higher CV risk markers [cIMT 0.86 (0.17) versus 0.77 (0.14) mm, *P* = 0.01; SCORE 4.3 (3.9) versus 1.1 (1.5), *P* < 0.0001], and higher Cys-C concentration [0.83 (0.24) versus 0.75 (0.21) mg/L, *P* = 0.04].

Significantly higher concentrations of SAA were found in patients with high disease activity (HDA) versus L/MDA (*P* < 0.0001) and in patients currently treated versus not treated with glucocorticoids (GCs) (*P* = 0.002) (Table 4). Significantly higher concentration of SAA was also observed in patients with advanced atherosclerosis when compared with those without atherosclerotic plaques (*P* = 0.04) and in patients with increased (≥1.0 mg/L) versus normal Cys-C level (*P* = 0.004) (Table 4).

The mean SAA concentration was significantly lower in women currently treated with biological DMARDs (*P* = 0.01) (Table 4). There was no such a correlation in men. Among female patients treated with biological DMARDs, anti-TNF inhibitors were used in 32 (94%) and rituximab was used in 2 cases.

### 3.4. Characteristics of Patients with Normal SAA Concentration

The normal SAA level (<10 mg/L) was observed in 11 patients (7.9%), 10 women, and 1 man. The mean concentration of SAA in this group was 6.4 (4.4) mg/L (range 0–9.9).

Patients with normal versus increased SAA concentration were characterized by lower concentration of CRP [11.4 (18.2) mg/L versus 20.2 (22.7), *P* = 0.03] and fibrinogen [4.1 (1.6) versus 4.8 (1.3) g/L, *P* = 0.05], lower WBC [6.0 (1.2) versus 8.1 (2.5) × 10^3^/*μ*L, *P* = 0.003], and higher QTc duration [373.8 (52.8) versus 332.2 (57.2) ms, *P* = 0.04]. There were no significant differences in methods of treatment between the two groups of patients.

## 4. Discussion

The major finding of the study was that serum SAA concentration was high above normal in the vast majority of RA patients. The normal SAA level was found only in <10% of patients, characterized by lower inflammatory burden and normal QTc duration. Elevated above normal SAA levels were found not only in patients with HDA, but also in those with L/MDA assessed with DAS28. This observation suggests that in spite of favorable clinical assessment, sustained inflammation persists in most of RA patients and may be connected with the disease complications.

In this study, we demonstrated a direct relationship between SAA and RA activity (with both clinical and laboratory parameters of activity). Significantly higher SAA was associated with the current treatment with GCs, which are commonly used in patients with HDA. Women treated with biological DMARDs had significantly lower SAA concentration than women treated only with synthetic DMARDs. These results confirm better therapeutic effects of biological DMARDs in comparison to synthetic DMARDs resulting in lower disease activity.

We also found relationships between SAA and CV risk factors. The mean SAA level was significantly higher in patients with plaques as a manifestation of advanced atherosclerosis. There was no relationship with cIMT value. In our observations, SAA level was inversely associated with QTc duration. Significantly higher mean SAA concentration was noticed in patients with increased Cys-C level as an early marker of chronic kidney disease (CKD).

The results of our study indicate that, in RA patients, SAA could be considered as a biomarker associated with both the current inflammatory disease activity and chronic complications, the risk of CV and renal involvement in the course of RA. Persistently elevated SAA concentration increases the risk of amyloidosis development due to deposition of amyloid proteins in different tissues and organs.

Our results are consistent with data in literature, which reported a strong correlation between SAA and RA activity [19]. The SAA level correlated with clinical disease activity (28-joint SJC) and was independently associated with 1-year radiographic progression in patients with inflammatory arthritis [6]. It has been demonstrated that SAA may be also produced by rheumatoid synovial tissue (but not in normal synovial membrane), inducing cell growth, angiogenesis, invasion and migration of cells, secretion of proinflammatory cytokines (TNF, IL-1, IL-6, and IL-8), chemokines, reactive oxygen species, and matrix metalloproteinases (MMP) [2, 6, 20, 21]. Therefore, SAA may play a pathogenic role locally in joint destruction and cartilage damage [20–22]. The correlation between SAA level and WBC may be associated with the reported function of SAA as a priming agent, rendering neutrophils more responsive to opsonized particles, as well as inducing chemotaxis and adhesion to endothelial cells and cellular migration [23, 24].

We noted an association between SAA level and the drug treatment. Significantly higher SAA levels were found in patients currently treated with GCs, which seems to be a paradoxical effect. However, GCs are commonly used in patients with HDA, presenting increased inflammatory parameters. On the other hand, it was reported that GCs induce extrahepatic SAA expression, which has been shown in tissue cultures [25] and confirmed in patients with chronic obstructive pulmonary disease [26]. That mechanism should also be taken into account considering patients with RA. Further prospective determinations of SAA are necessary to define an accurate answer. According to our knowledge, our report presents the first observation in RA patients.

We also found significantly higher SAA concentration in women not treated with biological DMARDs, which indicates more active disease in these cases. However, patients in our group received therapy with different biological DMARDs (one of the three TNF inhibitors or rituximab), which hinders a reliable assessment of relationship between SAA and biological treatment. In literature, there is no data on the association between SAA and rituximab. There are few reports on the effect of anti-TNF [27–31] and anti-IL-6 [31, 32] treatment on SAA concentration, dealing mostly with patients with amyloidosis, secondary to RA. The blockade of TNF prevents high systemic SAA concentrations that are largely driven by TNF. The effective and early DMARDs treatment works as the primary prevention of amyloidosis, as well as the secondary prevention in a subclinical phase of this complication [8]. However, even during anti-TNF therapy, excessive, extrahepatic expression of SAA, mediated by IL-6 and GCs, could probably be maintained in rheumatoid synovial tissue [25]. In this case, SAA may upregulate TNF itself and may be a potent upstream mediator of TNF, influencing response to existing anti-TNF therapy [6]. High level of SAA during anti-TNF treatment might serve as a biomarker of persistent synovial inflammation, resistant to the therapy currently used.

In our patients, the mean SAA concentration was significantly higher in men than in women, which may be related to significantly higher parameters of inflammation and unfavorable CV risk factors in men. According to literature, women have higher SAA levels than men, for various reasons, due to hormonal status, interaction with leptin, or different body composition [33, 34]. Men have larger intra-abdominal fat deposits, which produce significant amounts of cytokines (TNF, IL-6), even in larger quantity than subcutaneous adipose tissue [34].

We observed significantly higher SAA levels in patients with atherosclerotic plaques and increased Cys-C serum concentration, which indicates the risk of CV and renal complications. Our results are consistent with reports in literature. It has been demonstrated that cells within human atherosclerotic lesions (foam and endothelial cells, smooth muscles) could express SAA and release to circulation at the site of plaque rapture [35, 36]. No independent effect of SAA on cIMT value was observed in one study [37]. In literature, SAA is reported as a predictor of coronary artery disease and further coronary events, as well as biomarker of cerebrovascular disease [38, 39]. Cys-C has been reported as a prognostic marker of risk for death, CV, and kidney outcomes in elderly patients, with and without CKD [40].

In the whole group of our patients, the mean QTc duration was below 350 ms and was inversely correlated with SAA concentration. Intriguingly, in patients with normal SAA level, the mean QTc duration was normal. According to our knowledge, this is the first report on the association between SAA and short QTc, which should be taken into account, considering a risk of life-threating arrhythmias, especially in patients with high SAA concentration. Cardiovascular mortality is significantly increased in RA, with a risk of sudden death about twofold higher than in non-RA subjects [41, 42]. It has been suggested that this phenomenon may be associated with serious arrhythmias as a result of nonstructural cardiac alterations, such as reduced heart rate variability (HRV) or QT interval abnormalities [42]. Shortening of QTc, recognized with the value <350 ms, is associated with increased risk of atrial fibrillation with rapid ventricular tachycardia [43]. The study in patients with inflammatory arthritis demonstrated that high sensitivity CRP (hsCRP) was inversely correlated with HRV and directly correlated with QTc duration. The study population consisted mostly of patients with spondyloarthropathies and only 25% of RA patients of unknown disease activity [42]. It was a quite different population than the population presented in our study. The results may be different in highly selected sample of individuals. It is suggested that systemic inflammation may affect cardiac nervous system, irrespectively of other CV risk factors. It seems that a short QT interval does not by itself predict risk of life-threating arrhythmias but rather should be taken in context of each individual patient [44].

Our study has several strengths and limitations which should be taken into account. The strengths are as follows: (1) relatively large sample of consecutive patients with RA, treated in our department, (2) the detailed characteristics of patients, who have been considered in all aspects of RA pathology including several inflammatory, CV, metabolic parameters, and assessment of renal function, (3) the design of the study which was based on clinical practice, and (4) according to our knowledge being the first study which evaluates the potential association of high SAA level with QTc shortening, increasing the risk of serious arrhythmias and sudden death. There are also some limitations including the following: (1) our patients were treated with different biological DMARDs; that is why it is difficult to determine the exact association between SAA and effect of therapy; (2) ECG recording was performed routinely in every patient and QTc duration was assessed automatically, with no special procedure, and HRV was not assessed; and (3) we did not collect detailed information about the ongoing treatment, which may influence heart rate. As a consequence, further investigation is needed to confirm the observed relationship, in relation to concurrent treatment.

In our study, in patients with RA, the mean SAA concentration was highly above the normal value. Serum SAA levels strongly correlated with the activity of the disease and were significantly higher in patients with high disease activity. However, high SAA concentrations were observed also in patients with low or moderate disease activity, in spite of clinical improvement. Concurrent treatment with GCs was associated with higher SAA and therapy with biological DMARDs was associated with lower SAA concentration. The mean SAA level was inversely associated with QTc duration and markedly higher in patients with atherosclerotic plaques, emphasizing increased CV risk resulting from chronic inflammatory process. We also found significantly higher SAA levels in patients with increased Cys-C as a marker of CKD, emphasizing increased risk of renal disease.

## 5. Conclusions

High SAA concentration was strongly connected with the activity of the disease and the risk of CV and renal involvement in RA patients. Prospectively, repeated assessment of SAA may facilitate searching RA patients with persistent inflammation and increased risk of extra-articular complications, as well as amyloidosis development.

## Figures and Tables

**Table 1:** Baseline characteristics of 140 RA patients.

Variable	Results
Demographic variables	
Age (years)	50.3 (10.9) (range 18–76)
Gender (F/M)	111 (79.3%)/29 (20.7%)
RA related variables	
Disease duration (years)	10.1 (7.5) (range 0.5–33)
RA duration >10 years	64 (45.7%)
RF-IgM positivity	99 (70.7%)
ACPA positivity	116 (82.9%)
Erosions	117 (84%)
Extra-articular symptoms	58 (41.4%)
DAS28	4.52 (1.31) (range 1.9–8.5)
DAS28 ≤ 5.1	98 (70%)
DAS28 < 2.6	8 (5.7%)
M-HAQ	1.25 (0.62) (range 0–2.75)
Current MTX usage	82 (58.6%)
Current GC usage	108 (77.1%)
Current biologic therapy	36 (25.7%)

Values are mean (SD) or numbers (%).

RF-IgM: rheumatoid factor IgM; ACPA: anticitrullinated protein antibodies; DAS: Disease Activity Score; M-HAQ: Modified Health Assessment Questionnaire; MTX: methotrexate; GC: glucocorticoid.

**Table 2:** Clinical and laboratory variables in 140 RA patients.

Variable	Results
Clinical variables	
Systolic BP (mmHg)	126.6 (14.2) (range 90–160)
Diastolic BP (mmHg)	79.5 (9.3) (range 50–100)
BMI (kg/m^2^)	24.6 (3.8) (range 16–38.3)
SCORE (%)	1.7 (2.6) (range 0–12)
cIMT (mm)	0.79 (0.15) (range 0.43–1.2)
Plaques present	40 (28.6%)
QTc (ms)	335.5 (57.8) (range 186–487)
Laboratory variables	
CRP (mg/L)	19.5 (22.5) (range 0.1–116)
ESR (mm/h)	35.9 (24.3) (range 5–102)
Hemoglobin (g/dL)	12.5 (1.3) (range 9.2–16.4)
Total protein (g/dL)	7.1 (0.6) (range 4.2–8.4)
Albumin (g/dL)	4.2 (0.4) (range 2.3–5.0)
Fibrinogen (g/L)	4.8 (1.3) (range 2.0–9.0)
Total cholesterol (mg/dL)	200.1 (45.2) (range 98–365)
HDL-C (mg/dL)	59.9 (15.8) (range 32–115)
LDL-C (mg/dL)	117.4 (34.9) (range 38–245)
Triglycerides (mg/dL)	117.3 (55.5) (range 24–300)
Serum creatinine (mg/dL)	0.71 (0.23) (range 0.4–2.3)
MDRD-eGFR (mL/min/1.73 m^2^)	106.0 (26.7) (range 22.9–156)
Cystatin-C (mg/L)	0.77 (0.22) (range 0.42–1.66)
Serum amyloid A (mg/L)	327.0 (263.4) (range 0–733.2)

Values are mean (SD) or numbers (%).

BP: blood pressure; BMI: body mass index; SCORE: systemic coronary risk evaluation; cIMT: carotid intima-media thickness; CRP: C-reactive protein; QTc: corrected QT interval; ESR: erythrocyte sedimentation rate; HDL-C: high-density lipoprotein cholesterol, LDL-C: low-density lipoprotein cholesterol; MDRD-eGFR: estimated glomerular filtration by modification of diet in renal disease.

**Table 3:** Multiple linear regression analysis of associations between SAA concentration and laboratory as well as ECG parameters.

SAA/variables	*B*	*P* value
CRP	0.44	<0.0001
WBC	0.318	<0.0001
QTc	−0.21	0.004

SAA: serum amyloid A; CRP: C-reactive protein; WBC: white blood cell count; QTc: corrected QT interval.

**Table 4:** Significant differences in SAA concentration between groups of RA patients.

Variables	SAA (mg/L)	*P* value
Gender		
Male	433.6 (238.0)	0.01
Female	298.9 (263.6)	
Disease activity		
DAS28 > 5.1	478.3 (228.1)	<0.0001
DAS28 ≤ 5.1	261.5 (251.4)	
Current GC treatment		
Positive	361.1 (261.0)	0.002
Negative	213.0 (0.12)	
Carotid plaques		
Positive	388.3 (250.6)	0.04
Negative	302.2 (265.7)	
Cystatin-C		
≥1.0 mg/L	492.4 (229.2)	0.004
<1.0 mg/L	303.9 (260.4)	

Only in women		
Current biological DMARDs		
Negative	332.1 (266.5)	0.01
Positive	210.2 (237.8)	

Values are mean (SD).

DAS: disease activity score; GC: glucocorticoid.
